# Dynamic functional clot formation in patients undergoing endoscopic mucosal resection

**DOI:** 10.1002/jgh3.12306

**Published:** 2020-02-06

**Authors:** Bernd Froessler, Leonardo Zorron Cheng Tao Pu, Natalie Aboustate, Amanda Ovenden, Rajvinder Singh

**Affiliations:** ^1^ Department of Anaesthesia Lyell McEwin Hospital Adelaide South Australia Australia; ^2^ Discipline of Acute Care Medicine University of Adelaide Adelaide South Australia Australia; ^3^ Faculty of Health and Medical Sciences The University of Adelaide Adelaide South Australia Australia; ^4^ Department of Gastroenterology and Hepatology Nagoya University Graduate School of Medicine Nagoya Aichi Japan; ^5^ Robinson Research Institute Lyell McEwin Hospital Adelaide South Australia Australia; ^6^ Department of Gastroenterology and Hepatology Lyell McEwin Hospital Adelaide South Australia Australia

**Keywords:** electric burn, endoscopic mucosal resection, fibrinolysis, hemorrhage, point‐of‐care systems

## Abstract

**Background and Aim:**

Electric burns can induce fibrinolytic activity. Endoscopic mucosal resection (EMR) is a minimally invasive technique utilizing electrocautery for resection of dysplastic lesions from the gastrointestinal tract. EMR's main complication is clinically significant postendoscopic bleeding. Currently, no studies have investigated the impact of electrocautery during EMR on the coagulation cascade by viscoelastic hemostatic assay.

**Methods:**

Thrombelastometry was performed and plasminogen levels were measured on blood samples taken prior to EMR, within an hour following the procedure and 2 days post‐EMR. Data were natural log‐transformed and analyzed using repeated‐measure analysis of covariance (ANCOVA) accounting for age, sex, body mass index (BMI) and site of EMR.

**Results:**

Plasminogen levels decreased post‐EMR (*P* = 0.001) and then increased 2 days post‐EMR (*P* < 0.018). FIBTEM A10 and Maximum Clot Firmness, and EXTEM maximum lysis decreased an hour following EMR (*P* < 0.05 for all). These three measurements then increased 2 days post‐EMR (*P* < 0.01 for all). There were no significant differences in thrombelastometry or plasminogen measures according to sex, age, BMI, or site of EMR. One patient experienced clinically significant postendoscopic bleeding at one‐week post‐EMR, with substantially decreased FIBTEM A10 and Maximum Clot Firmness at 2 days post‐EMR.

**Conclusions:**

Decreased post‐EMR plasminogen corresponds with reduced clot firmness and enhanced lysis affecting clot quality, strength, and stability. While further investigation in a larger sample is required to confirm the overall risk of clinically significant postendoscopic bleeding and mechanisms for plasminogen activation; this study highlights the potential utility of thrombelastometry in assessing fibrinolytic activity during EMR.

## Graphical abstract

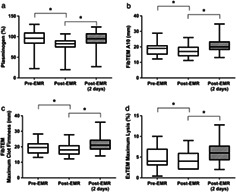

Decreased post‐EMR plasminogen corresponded with decreased clot firmness and enhanced lysis. We suspect plasminogen activation may lead to fibrinogen utilization and our results highlight the possible utility of incorporating point of care testing in assessing fibrinolytic activity in EMR.

## Introduction

Approximately, 16 000 colonic cancers are diagnosed in Australia each year.^1^ Most of these patients undergo surgery, which is associated with significant morbidity and mortality, particularly in the elderly.^2^ Recently, advances in endoscopic mucosal resection (EMR) have resulted in its establishment as a cost‐effective and minimally invasive technique for the removal of precancerous and early cancerous gastrointestinal lesions.^3^ EMR can be performed as an ambulatory procedure in specialized Gastroenterology units. A primary risk factor of EMR is clinically significant post‐EMR bleeding (CSPEB).^4^ Though the underlying cause for CSPEB remains unclear, one study has seemingly prevented delayed bleeding in a small sample of patients undergoing EMR by using submucosal injection of autologous platelet‐rich plasma.^5^ A randomized controlled trial comparing underwater EMR to conventional EMR did not demonstrate a significant difference in postoperative hemorrhage.^6^ Clot formation and breakdown are in delicate balance that can be disrupted by congenital or acquired conditions.^7^ Previous studies in other settings have demonstrated that electrosurgery and burns can cause plasminogen activation and fibrinolysis.^8^, ^9^ Viscoelastic hemostatic assays (VHA) rapidly assess the hemostatic system in whole blood, reflecting clot initiation, firmness and stability, and fibrinolytic activity. This provides a useful tool for clinicians that helps predict the risk of bleeding and need for targeted therapeutic intervention.^10^, ^11^ We therefore sought to examine whether electric burns imposed by EMR increased fibrinolytic activity and whether thrombelastometry could be used as a utility in identifying such risk in patients undergoing EMR.

## Material and methods

Patients undergoing ‘piecemeal’ EMR for dysplastic gastrointestinal lesions at the Lyell McEwin Hospital, an Australian endoscopy center, between July 2017 and January 2018 were consecutively invited to participate in the study. All eligible patients had been referred to the center with large lesions (>10 mm) without endoscopic features of invasive cancer. The size of the lesion was estimated by the endoscopist performing the procedure. The study was registered with The Australian New Zealand Clinical Trials Registry (ACTRN12617000530325). Participants provided informed, written consent to participate in the study as approved by the Northern Adelaide Health Network's Human Research Ethics Committee (HREC/17/TQEH/66). While existing literature suggests that up to 7% of patients in the EMR population experience CSPEB, no current studies have investigated VHA parameters by Rotational Thrombelastometry (ROTEM) in this context. As a purely observational study, we therefore aimed for a standard pilot sample of 20 participants in an effort to inform power calculations for future larger studies. Whole blood was collected into a standard draw citrate Vacuette® tubes (3.5 mL) at three time points: prior to the procedure (pre‐EMR), within an hour following the procedure (post‐EMR) and 2 days following procedure (2‐days post‐EMR).

Thrombelastometry (ROTEM supplier to Instrumentation Laboratory, Bedford, USA) including EXTEM (measuring standard clot formation activating the extrinsic pathway) and FIBTEM tests (measuring the contribution of fibrinogen to clot formation and strength by adding a Platelet inhibitor), and plasminogen assays were undertaken by the hospital's pathology provider, SA Pathology.

Lysis values (%) were taken at 60 min (LY60) to illustrate clot reduction from maximum clot formation (MCF). The EXTEM assay uses a tissue factor to activate the extrinsic pathway and the FIBTEM assay provides an indicator of clot tracing that reflects fibrinogen concentration and function, by adding a Platelet inhibitor. Plasminogen was measured through mass‐spectrometry of plasma samples.

Patient characteristics are reported as mean and standard deviation, or frequency and percentages. Our laboratory data were nonparametric and therefore, natural log transformations were performed on ROTEM and plasminogen measures prior to statistical analysis. These data are reported as the median, and 25th and 75th centiles. Repeated measures ANCOVAs were conducted on ROTEM and plasminogen parameters adjusting for sex, age, body mass index (BMI), and site of EMR. An alpha level of 0.05 was considered statistically significant.

## Results

Twenty‐three participants were sampled pre‐EMR and post‐EMR, with 21 individuals returning for sampling 2 days post‐EMR. Participant characteristics are reported in Table 1 and results summarized in Table 2.

**Table 1:** Clinical characteristics of participants undergoing EMR (*n* = 23) expressed as frequencies, unless otherwise indicated

Age (mean years ±SD)	67 ± 11
Sex	
Male	11 (48%)
Female	12 (52%)
BMI (mean kg/m^2^ ± SD)	28.4 ± 6
Site of resection	
Duodenum	4 (17%)
Esophagus	2 (9%)
Colon	15 (65%)
GEJ	1 (4%)
Rectum	1 (4%)

GEJ, gastroesophageal junction; BMI, body mass index.

**Table 2:** Plasminogen and thrombelastometry measures across three time points sampled in participants undergoing EMR

	Pre‐EMR	Post‐EMR	2‐day post‐EMR	*P*‐value
Plasminogen (%)	98 (86–113)	85 (77–91)	96 (88–111)	0.001*
EXTEM CT (s)	64 (60–67)	62 (58–68)	67 (61–72)	0.252
EXTEM CFT (s)	73 (63–82)	78 (70–96)	71 (64–87)	0.086
EXTEM A10 (mm)	58 (56–61)	57 (51–60)	61 (55–64)	0.131
EXTEM MCF (mm)	65 (63–69)	64 (61–68)	68 (63–71)	0.098
EXTEM ML (%)	4 (3–7)	4 (2–6)	6 (5–7)	0.001*
FIBTEM A10 (mm)	19 (15–21)	17 (14–20)	20 (18–23)	<0.001*
FIBTEM MCF (mm)	20 (16–23)	18 (15–21)	21 (19–24)	0.001*

Data are expressed as median (25th–75th centile) where *P < 0.05.

**Figure 1:**
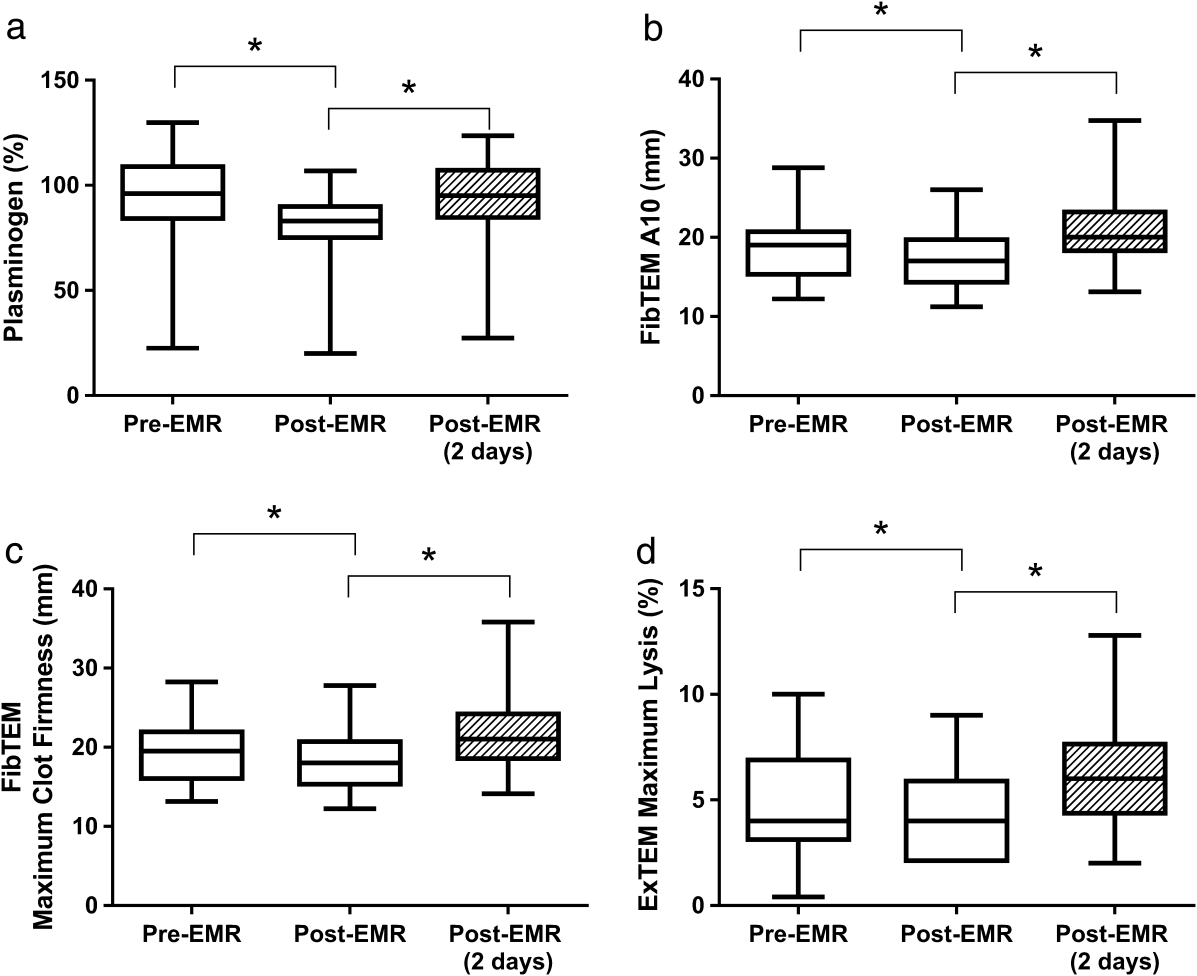
Measures of fibrinolysis including EX‐ML (a), F‐A10 (b), F‐MCF (c) and plasminogen (d); across 3 time points in participants undergoing EMR. Data are expressed as median (25th–75th centile), where *P < 0.05.

Plasminogen (% normal plasma) was significantly different from baseline across the sampled time points (F[2,38] = 8.635, *P* = 0.001), where post‐hoc pairwise comparisons indicated plasminogen decreased post‐EMR (*P* = 0.001), and subsequently increased between 2 days post‐EMR compared to measures taken post‐EMR (*P* = 0.018; Fig. 1a). FIBTEM A10 (mm) and MCF, and EXTEM ML followed a similar pattern of temporal expression. Specifically, FIBTEM A10 (mm) was significantly different across the sampled time points (F[2,38] = 9.655, *P* < 0.001), with pairwise comparisons indicating it decreased post‐EMR (*P* = 0.033) and then increased 2 days post‐EMR compared to post‐EMR measures (*P* < 0.001; Fig. 1b); FIBTEM MCF (mm) changed significantly across the time points (F[2,34] = 7.965, *P* = 0.001), with pairwise comparisons indicating it decreased post‐EMR (*P* = 0.048) and then increased 2 days post‐EMR compared to post‐EMR measures (*P* = 0.001; Fig. 1c), and EXTEM ML (%) changed significantly across the time points (F[2,36] = 7.873, *P* = 0.001), with pairwise comparisons indicating it decreased post‐EMR (*P* = 0.045) and then increased 2 days post‐EMR compared to post‐EMR measures (*P* = 0.001; Fig. 1d). EXTEM CT, CFT, A10, and MCF remained unchanged across the sampled time points.

Of note, one of our participants (an 89‐year‐old female) presented to the emergency department with a CSPEB 8 days post‐EMR (resection of a 6–7 cm circumferential tubulovillous adenoma of the rectum). She experienced four episodes of sudden‐onset bright red and painless PR bleeding while at home and was unable to quantify the amount of blood lost. She was hemodynamically stable, but her hemoglobin decreased from 118 to 97 g/L. The patient was admitted under Gastroenterology to our High Dependency Unit for monitoring. Computer tomography with iodinated intravenous contrast of the abdomen/pelvis could not determine the site of active bleeding. A flexible sigmoidoscopy was undertaken and showed a large ulcer at the rectal EMR site, with a small adherent clot at its base that was not actively bleeding. The patient's anemia was managed with a two‐unit packed red blood cell transfusion and an iron infusion. There was no evidence of coagulopathy from standard laboratory testing, with normal Prothrombin time, INR, and aPTT present. The patient's thrombelastometry at readmission revealed no significant changes on EXTEM; however, her FIBTEM values decreased substantially from her 2‐day post‐EMR follow‐up. Specifically, her FIBTEM A10 had changed from 24 to 16 mm and FIBTEM MCF from 26 to 16 mm. The patient remained stable throughout her admission, no further PR bleeding occurred, and she was discharged home with planned General Practitioner and Gastroenterology follow‐up.

## Discussion

This is the first study to characterize ROTEM parameters that may contribute to the development of CSPEB in EMR patients. CSPEB can precede major bleeding, and both are potentially associated with morbidity, mortality, and costly hospital readmissions. Acquired fibrinolysis can occur under a number of conditions, including thermal injury, leukemia, trauma, and gastro‐intestinal cancers.^12^, ^13^, ^14^ Our pilot cohort demonstrated a decrease in plasminogen levels post‐EMR occurring alongside decreased clot firmness (FIBTEM A10, MCF), and enhanced lysis (EXTEM ML). This may present evidence of plasminogen activation and therefore, increased post‐EMR bleeding risk. Notably, samples taken 2 days following the procedure showed recovery of these measures to levels that were comparable to samples taken prior to EMR. As others have reported a correlation between plasma fibrinogen and FIBTEM MCF, our observed increase in MCF may form part of an acute‐phase response after tissue injury, surgery, inflammation, or infection: which is often observed 2–3 days postoperatively.^15^

The current pilot study is small and observational, and therefore carries some limitations. Firstly, although our analysis attempted to adjust for sex, age, BMI, and resection location, these factors likely remain influential. Secondly, despite the validation of thromboelastometry and its utilization in many clinical areas as a point of care device for fast detection of hemostatic disturbances,^16^ the incorporation of more sensitive markers of fibrinolysis, such as tissue‐type plasminogen activator, plasmin–antiplasmin complex, fibrin degradation products, or levels of inhibitors of fibrinolysis (alpha2‐Antiplasmin, plasminogen activator inhibitor 1) should be incorporated in future analyses to validate its utility.^17^ Additionally, the inclusion of the APTEM test could have provided us with information on whether antifibrinolytic drug administration could have impacted on our results. Overall, a larger sample size to adjust for these factors is therefore required to reinforce our findings.

All patients in our cohort underwent piecemeal EMR for large lesions. However, variations such as time of procedure, lesion size, and presence of intraprocedural bleeding may affect the degree of the electrocautery fibrinolysis effect. The small numbers of this initial investigation did not allow for us to control and investigate all potential factors influencing the amount of electrocautery used. Future research considering these effects is therefore warranted.

The rate and extent of fibrinolysis is affected by clot properties, where weaker clots carry an increased risk of clinically significant bleeding.^18^ Our work concerning thromboelastometry is therefore critical in beginning to understand hemostatic disturbances that precede CSPEB in EMR patients. As such, local fibrinogen administration or systemic antifibrinolytic intervention should be explored and may offer an effective intervention in preventing post‐EMR bleeding.

## Conclusion

Our study found that decreased post‐EMR plasminogen corresponded with decreased clot firmness and enhanced lysis. In this context, we suspect plasminogen activation may lead to fibrinogen utilization and our results highlight the possible utility of incorporating point of care testing in assessing fibrinolytic activity in EMR. The underlying mechanisms of plasminogen activation and the potential for protective intervention require further investigation.

## References

[1] Welfare AIoHa . Cancer in Australia 2019. Australian Institute of Health and Welfare, Canberra, 2019.

[2] McNicol L , Story DA , Leslie K *et al* Postoperative complications and mortality in older patients having non‐cardiac surgery at three Melbourne teaching hospitals. Med. J. Aust. 2007; 186: 447–52. 17484705 10.5694/j.1326-5377.2007.tb00994.x

[3] Moss A , Bourke MJ , Williams SJ *et al* Endoscopic mucosal resection outcomes and prediction of submucosal cancer from advanced colonic mucosal neoplasia. Gastroenterology. 2011; 140: 1909–18. 21392504 10.1053/j.gastro.2011.02.062

[4] Burgess NG , Metz AJ , Williams SJ *et al* Risk factors for intraprocedural and clinically significant delayed bleeding after wide‐field endoscopic mucosal resection of large colonic lesions. Clin. Gastroenterol Hepatol. 2014; 12: 651–61. e1–3. 24090728 10.1016/j.cgh.2013.09.049

[5] Lorenzo‐Zuniga V , de Vega VM , Bartoli R *et al* Submucosal injection of platelet‐rich plasma in endoscopic resection of large sessile lesions. World J. Gastrointest. Endosc. 2018; 10: 348–53. 30487945 10.4253/wjge.v10.i11.348 PMC6247099

[6] Yamashina T , Uedo N , Akasaka T *et al* Comparison of Underwater vs Conventional Endoscopic Mucosal Resection of Intermediate‐Size Colorectal Polyps. Gastroenterology. 2019; 157: 451–61.e2. 30981791 10.1053/j.gastro.2019.04.005

[7] Grottke O , Fries D , Nascimento B . Perioperatively acquired disorders of coagulation. Curr. Opin. Anaesthesiol. 2015; 28: 113–22. 25734869 10.1097/ACO.0000000000000176 PMC4418784

[8] Shubina TA , Lyutova LV , Karabasova MA , Mynbaev OA , Andreenko GV . Coagulation and fibrinolysis in rats after surgery with monopolar electrical scalpel. Bull. Exp. Biol. Med. 2000; 130: 917–20. 11177281

[9] Gibson B , Moore‐Lotridge S , Mignemi N , Hawley G , Oelsner W , Schoenecker J . The consumption of plasminogen following severe burn and its implications in muscle calcification. FASEB J. 2017; 31: 390.4–4.

[10] Levi M , Hunt BJ . A critical appraisal of point‐of‐care coagulation testing in critically ill patients. J. Thromb. Haemost. 2015; 13: 1960–7. 26333113 10.1111/jth.13126

[11] Franchini M , Mengoli C , Cruciani M *et al* The use of viscoelastic haemostatic assays in non‐cardiac surgical settings: a systematic review and meta‐analysis. Blood Transfus. 2018; 16: 235–43. 29517967 10.2450/2018.0003-18 PMC5919835

[12] Okajima K , Kohno I , Soe G , Okabe H , Takatsuki K , Binder BR . Direct evidence for systemic fibrinogenolysis in patients with acquired alpha 2‐plasmin inhibitor deficiency. Am. J. Hematol. 1994; 45: 16–24. 8250008 10.1002/ajh.2830450104

[13] Meijer K , Smid WM , Geerards S , van der Meer J . Hyperfibrinogenolysis in disseminated adenocarcinoma. Blood Coagul. Fibrinolysis. 1998; 9: 279–83. 9663712 10.1097/00001721-199804000-00010

[14] Lavrentieva A , Kontakiotis T , Bitzani M *et al* Early coagulation disorders after severe burn injury: impact on mortality. Intensive Care Med. 2008; 34: 700–6. 18193192 10.1007/s00134-007-0976-5

[15] Reinhart WH . Fibrinogen—marker or mediator of vascular disease? Vasc. Med. 2003; 8: 211–6. 14989564 10.1191/1358863x03vm494ra

[16] Spiel AO , Mayr FB , Firbas C , Quehenberger P , Jilma B . Validation of rotation thrombelastography in a model of systemic activation of fibrinolysis and coagulation in humans. J. Thromb. Haemost. 2006; 4: 411–6. 16420574 10.1111/j.1538-7836.2006.01715.x

[17] Saes JL , Schols SEM , van Heerde WL , Nijziel MR . Hemorrhagic disorders of fibrinolysis: a clinical review. J. Thromb. Haemost. 2018; 16: 1498–509. 10.1111/jth.14160 29847021

[18] Pieters M , Wolberg AS . Fibrinogen and fibrin: An illustrated review. Res. Pract. Thromb. Haemost. 2019; 3: 161–72. 31011700 10.1002/rth2.12191 PMC6462751

